# Inhibition of 11βHSD1 with the S-phenylethylaminothiazolone BVT116429 increases adiponectin concentrations and improves glucose homeostasis in diabetic KKA^y ^mice

**DOI:** 10.1186/1471-2210-8-3

**Published:** 2008-02-12

**Authors:** Maj Sundbom, Christina Kaiser, Eva Björkstrand, Victor M Castro, Catarina Larsson, Göran Selén, Charlotte Söderberg Nyhem, Stephen R James

**Affiliations:** 1 Biovitrum, S-112 76 Stockholm, Sweden; 2 Biolipox, S-171065 Solna, Sweden; 3 Department of Molecular Medicine and Surgery, Karolinska Institute, Stockholm, Sweden

## Abstract

**Background:**

A substantial body of evidence indicates that reduced plasma adiponectin levels may be key in the development of insulin resistance, type 2 diabetes and the metabolic syndrome. Glucocorticoids decrease the levels of adiponectin in animals and humans. Cortisone is transformed to its active form cortisol, via 11β-hydroxysteroid dehydrogenase (HSD) type 1. This study sought to ascertain if inhibition of 11β HSD1 with a new selective inhibitor, BVT116429, affects the concentrations of circulating adiponectin with concomitant effects on glucose homeostasis in diabetic mice.

**Results:**

KKA^y ^mice were treated with BVT116429 (3, 10, 30 mg/kg), rosiglitazone (5 mg/kg) or vehicle once daily for ten days. Plasma adiponectin levels rose in mice treated with BVT116429 and this was found to be both the hexameric and the high molecular weight multimeric forms of adiponectin. Seven days of treatment with the 11β HSD1-inhibitor BVT116429 decreased basal insulin levels but no changes in glucose tolerance were seen. After ten days of treatment, fasting blood glucose level was decreased by BVT116429 comparable to the effects of rosiglitazone. Another 11β HSD1 inhibitor, BVT2733, improved HbA1c but had no effect on adiponectin.

**Conclusion:**

Inhibition of 11β HSD1 can be expected to be beneficial for treating the pathology of type 2 diabetes mellitus. The differences seen in adiponectin between BVT116429 and BVT2733 could be explained by different pharmacodynamics exerted by the compounds in different tissues in the body. Increases in adiponectin concentrations may be an integral component in the mechanism of action of this new11β HSD1 inhibitor and may be a useful marker of efficacy during the clinical development of 11β HSD1 inhibitor compounds.

## Background

Type 2 diabetes and Cushing's syndrome have similar phenotypes, including visceral obesity, dyslipidemia, hyperglycemia and insulin resistance [1]. In the case of Cushing's syndrome, the patients have elevated circulating glucocorticoids whereas patients with type 2 diabetes have normal circulating levels but higher intracellular levels [2]. The concentration of active glucocorticoids (cortisol, corticosterone) formed at the cellular level is regulated by the degree of reductase activity of the 11β-hydroxysteroid dehydrogenase (HSD) type 1 enzyme [3].

Glucocorticoids have been shown to decrease the levels of adiponectin in animal models [4,5] and in humans [6]. Adiponectin is a hormone secreted exclusively from adipose tissue [7], the concentrations of which are positively correlated with a favorable metabolic phenotype in humans [8]. It circulates in serum as a basic trimer, a hexamer, and larger multimeric structures [9]. The full-length protein influences hepatic gluconeogenesis whilst the globular domain of adiponectin stimulates β-oxidation in muscle. These effects are mediated by the AdipoR2 (liver) and AdipoR1 (muscle) receptors respectively [10]. Recently it was shown that the APPL1 adaptor protein binds to the intracellular domain of adiponectin receptors and confers some of adiponectin's actions [11], and thus may be an important mediator of adiponectin-dependent insulin sensitization in skeletal muscle. The mRNA expression of adiponectin is reduced in adipose tissue in obese mice and humans [12] although the picture differs somewhat depending on fat depot. Adiponectin is not only decreased in obese individuals but also in type 2 diabetic patients [7] and a low level is a risk factor for developing this disease [13]. Physiological doses of adiponectin improve insulin sensitivity in db/db and KKA^y ^mice, two mouse models of type 2 diabetes, partly by decreasing triglycerides (TG) in liver and muscle tissue [14]. Treatment with thiazolidinediones increases the expression and secretion of adiponectin in both rodents and humans [15,16], indicating that changes in this adipokine may be integral to the therapeutic effects of thiazolidinediones [17,18].

Transgenic mice overexpressing 11β HSD1-selectively in adipose tissue develop type 2 diabetes and visceral obesity [4], in contrast to the 11β HSD1 knockout mouse, which displays a favorable phenotype with normoglycemia and normal weight [5,19,20]. This animal resists diet-induced obesity, has improved glucose and insulin tolerance and demonstrates favorable changes in cytokine expression, including a doubling in adiponectin. Another transgenic mouse overexpressing HSD type 2 in adipose tissue, and thereby inhibiting corticosterone exposure intracellularly, also had higher expression of adiponectin than its wild type counterparts when fed a high fat diet [21]. Various inhibitors of 11β HSD1 have been disclosed, with multiple favourable effects on the metabolic phenotype. In addition to increased insulin sensitivity and glucose tolerance [22-26], anti-obesity effects such as reduced food intake, body weight gain and reduced percentage body fat have been reported [24,25]. Furthermore, atherosclerotic plaque lesions in apoE knockout mice were improved after treatment with 11β HSD1 inhibitors [25]. However, despite favourable effects on the insulin-sensitising adipokine adiponectin in genetic models of 11β HSD1 inhibition and corticosterone reduction, no such analysis has been reported in pharmacological models. Substance BVT116429 inhibited11β HSD1 in adipose tissue. It has good bioavailability (75% is free from plasma protein binding) and low clearance, resulting in high Cmax values [27]. When tested in C57Bl6 mice, the compound displayed excellent pharmacodynamics in adipose tissue, inhibiting 11β HSD1 activity by 90%, 2 and 6 hours after dosing (30 mg/kg) [27]. The aim of this study was to investigate if inhibition of 11β HSD1 in adipocytes with the specific inhibitor BVT116429 affects the concentrations of adiponectin with concomitant impact on glucose homeostasis in diabetic mice. We compared the effects with BVT2733 [23], another 11β HSD1 inhibitor shown to exert its effects through alteration of glucose handling in the liver.

## Results

### Potency and pharmacodynamic activity of 11β HSD1 inhibitor BVT116429

**Figure 1:**
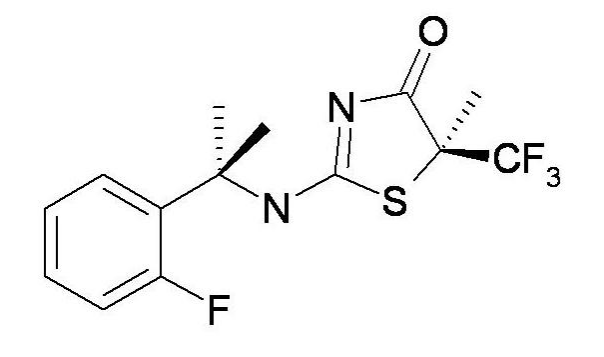
Structure of BVT116429.

**Figure 2:**
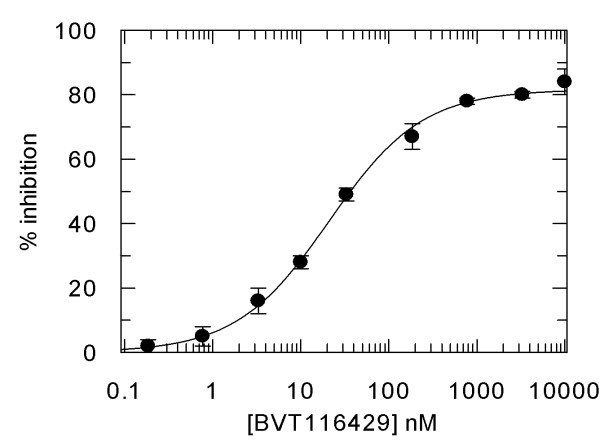
**Inhibition curve for human 11β HSD1 by increasing concentrations of BVT116429**. Error bars are standard deviations of triplicate determinations. The solid line is a fit of the data to the equation y = Range/(1+(x/IC_50_)^s^) where range is the uninhibited value and s the slope factor.

**Figure 3:**
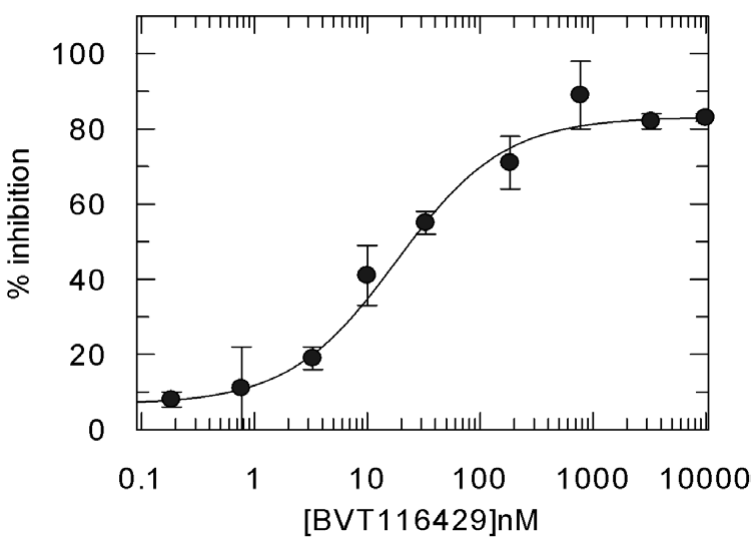
**Inhibition curve for production of cortisol from cortisone by primary human adipocytes**. Terminally differentiated adipocytes were incubated in the presence of cortisone and increasing concentrations of BVT116429 and cortisol concentrations determined from the cell supernatants. Data are means of triplicate determinations with standard deviations indicated. The solid line is a fit of the data to the same equation as in Fig 2.

BVT116429 ((S)-2-((S)-1-(2-fluorophenyl)ethylamino)-5-methyl-5-(trifluoromethyl)thiazol-4(5H)-one (Fig. 1) is a potent reversible competitive inhibitor of human and mouse 11β HSD1 with > 250 fold selectivity over human 11β HSD2. Inhibitory constants derived from in vitro inhibition assays using purified recombinant mouse and human 11β HSD1 were 36 and 29 nM, respectively (average from repeated assays performed in triplicate). Representative data for human the enzyme is shown in figure 2. BVT116429 had no significant effect on a panel of 80 unrelated proteins and ion channels (data not shown). To determine the effect of BVT116429 in cells, human primary adipose cells were treated with increasing concentrations of compound and the ability of the cells to convert cortisone to cortisol was assessed using a cortisol ELISA assay. Repeated assays showed that BVT116429 inhibited intracellular 11β HSD1 with an IC50 of 26 nM (Fig. 3). To understand the degree of 11β HSD1 inhibition in liver from mice dosed with BVT116429 at 30 mg/kg, the liver was excised 2 h post last dose, minced and exposed to cortisone ex vivo for 30 minutes. Data showed that the compound inhibits conversion of cortisone to cortisol by 11β HSD1 in liver such that two hours post-dosing, liver 11β HSD1 activity was reduced by 35% (2.12 ± 0.40 ng cortisol formed/mg/30 min in vehicle and 1.36 ± 0.25 ng cortisol formed/mg/30 min in BVT116429). This contrast with the compounds greater effects in adipose tissue (90% 6 h post dosing) [27]. Thus, BVT116429 displays significant inhibitory activity against the enzyme in key target organs after oral dosing in C57/Bl6J mice with especially high activity in adipose tissue.

### Effect of BVT116429 treatment on plasma glucose

**Figure 4:**
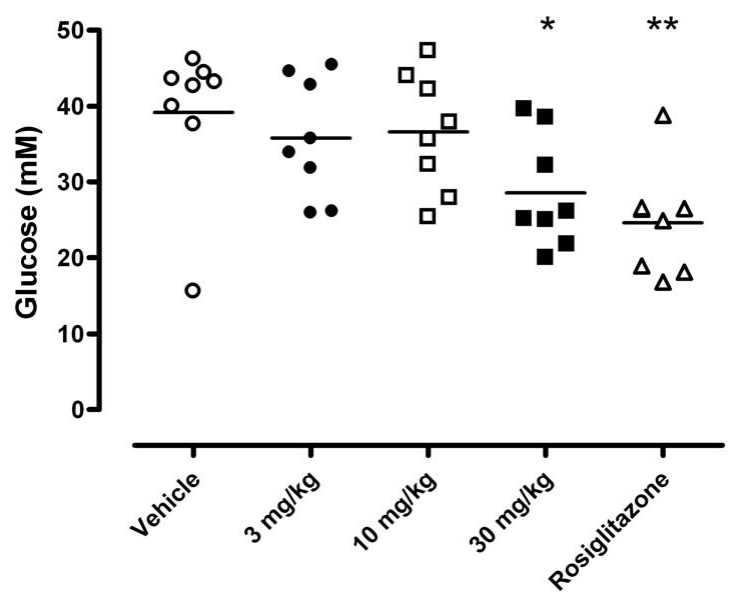
**Effects of BVT116429 on plasma glucose in KKA**^y^**mice**. The effect after 10-days treatment with vehicle (white circles), BVT116429 3 mg/kg (black circles), BVT116429 10 mg/kg (white squares), BVT116429 30 mg/kg (black squares) or rosiglitazone (white triangles) on plasma glucose. *p < 0.05 and **p < 0.01 when compared with vehicle.

To test the effects of BVT116429 on the diabetic phenotype of KKA^y ^mice, male mice were treated with compound for 10 days as described in the Methods section. Fasting glucose was reduced in animals treated with 30 mg/kg BVT116429, an effect that was comparable to that of rosiglitazone (Fig. 4).

### Effect of BVT116429 treatment on adiponectin in vivo and in vitro

**Figure 5:**
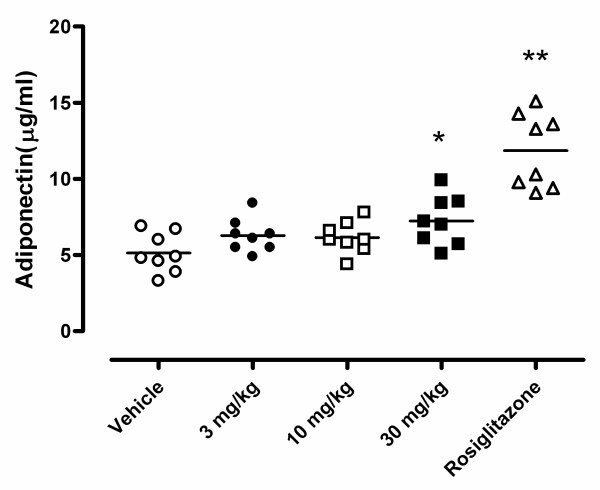
**Effects on plasma adiponectin in KKA^y ^mice**. The effect after 10-days treatment with vehicle (white circles), BVT116429 3 mg/kg (black circles), BVT116429 10 mg/kg (white squares), BVT116429 30 mg/kg (black squares) or rosiglitazone (white triangles) on plasma adiponectin. * *p *< 0.05, ** *p *< 0.01 when compared with vehicle.

**Figure 6:**
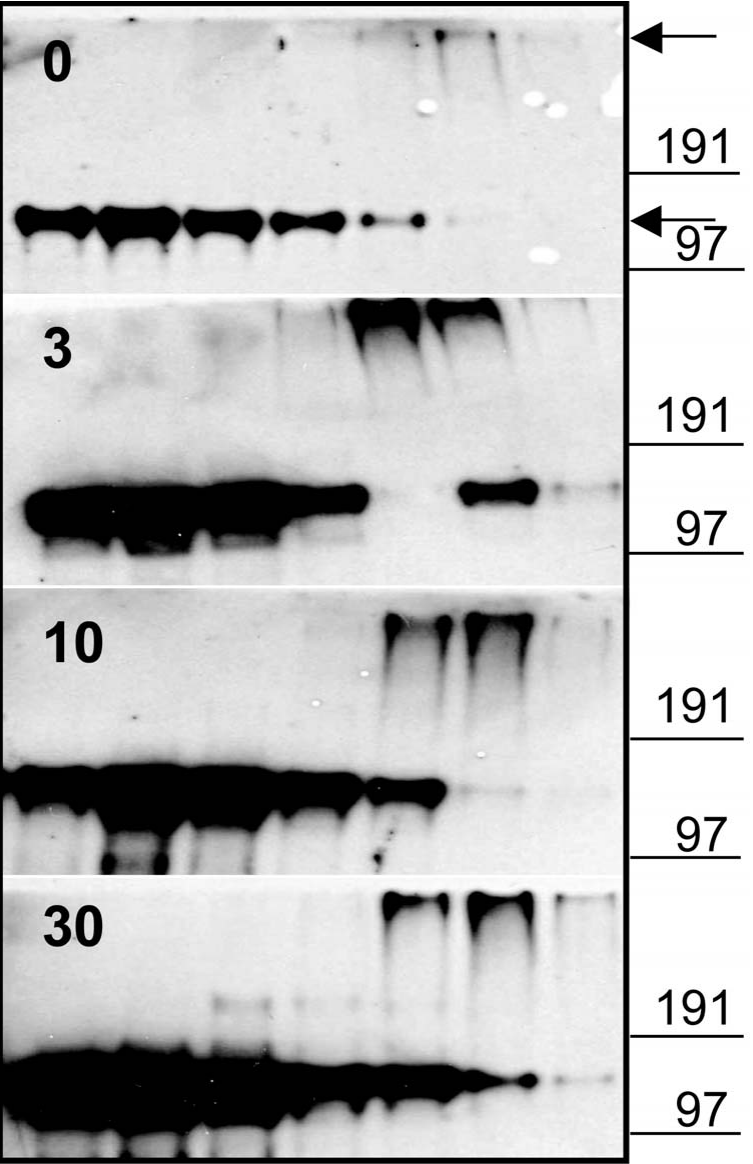
**Proportion of high and medium molecular weight adiponectin in KKA^y ^mice**. Velocity sedimentation analysis of serum from KKA^y ^mice treated with the indicated amounts of compound BVT116429 or vehicle was performed. This is a representative immunoblot showing the changes of the different adiponectin forms. Several independent analyses were performed giving similar results. The two main adiponectin complexes, HMW and MMW bands, are indicated by arrows and molecular mass markers are shown.

Different transgenic mouse models with altered 11β HSD1 activity or glucocorticoid concentrations demonstrate elevated adiponectin concentrations. For this reason, and considering the apparently greater effects of BVT116429 on 11β HSD1 activity in adipose tissue, we measured adiponectin in KKA^y ^mice treated with BVT116429. Total adiponectin levels were elevated in KKA^y ^mice after treatment with both BVT116429 (30 mg/kg) and rosiglitazone compared with control (Fig. 5). In view of recent findings that the distribution of different adiponectin complexes is critical in determining insulin sensitivity [9], we decided to investigate the complex composition of adiponectin in serum from the KKA^y ^mice. The most prominent adiponectin complex in KKA^y ^mice was the hexamer (MMW). Both the hexameric and the high molecular weight (HMW) multimeric forms were increased after treatment with BVT116429 (Fig. 6) and rosiglitazone (data not shown). In order to understand the mechanistic interplay between 11β HSD1 inhibition and a concomitant increase of adiponectin levels better, we studied human primary adipocytes in culture. Terminally differentiated human adipocytes were treated with BVT116429 for 48 hours in the presence of cortisone. Adiponectin concentrations released into the culture medium increased by 1.5-fold, from 13.6 ng/ml in the absence of compound to 20.1 ng/ml in its presence (mean values from pooled samples of sextuplicate determinations). Thus, inhibition of 11β HSD1 activity in adipocytes is able to increase adiponectin production, suggesting that the effect seen in intact mice is a direct one on the adipose mass.

### Effect of BVT116429 on glucose tolerance and insulin sensitivity

**Figure 7:**
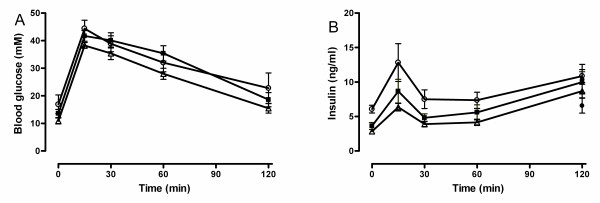
**Glucose tolerance test after treatment with BVT116429 in KKA^y ^mice**. The effect of vehicle (white circles), BVT116429 30 mg/kg (black squares) or rosiglitazone (white triangles) on glucose tolerance test. Plasma glucose (A) and insulin (B) are shown in response to 2 g/kg of glucose. Data are expressed as means ± SEM.

Glucose excursions after a glucose challenge in oGTT were not altered by either rosiglitazone or BVT116429, indicating no effect on glucose tolerance in these animals (Fig. 7A). The insulin curve is shown in figure 7B. Basal plasma insulin level was decreased for both rosiglitazone (2.85 ± 0.29 mg/ml, p < 0.01) and BVT116429 (30 mg/kg, 3.63 ± 0.29 ng/ml, p < 0.01) compared with vehicle (6.07 ± 0.55 ng/ml) indicating increased insulin sensitivity already after seven days of treatment.

To examine more specifically the effects of BVT116429 on insulin sensitivity, an insulin tolerance test was performed in male KKA^y ^mice. Animals were treated with BVT116429 for seven days prior to the test. After an overnight fast, animals were given an intraperitoneal bolus of insulin (1 U/kg) and blood glucose was followed for two hours. When fitted to a single exponential equation, the glucose elimination rate constant (min^-1 ^*1000) were 2.0 ± 0.6 (vehicle), 2.5 ± 1.0 (3 mg/kg BVT116429), 4.2 ± 0.9 (10 mg/kg BVT116429), 3.5 ± 0.7 (30 mg/kg BVT116429) and 5.0 ± 0.9 (5 mg/kg rosiglitazone). The apparent increased elimination rate constant did not however show statistical significance (p = 0.07, F-test).

### Euglycemic hyperinsulinemic clamp

To examine the effects of BVT116429 at the level of the liver more specifically, a euglycemic hyperinsulinemic clamp was performed in male KKA^y ^mice. Animals were treated once daily with 30 mg/kg BVT116429 or vehicle for eight days, starting three days after surgery to implant a catheter in the right jugular vein. After a 6 hour fast, the clamp was performed as described in the Methods section. BVT116429 had no effect on hepatic glucose production. Basal endogenous glucose production was 33.1 ± 9.5 (mg/kg.min) and 36.3 ± 12.8 (mg/kg.min), and after insulin infusion 23.7 ± 6.0 (mg/kg.min) and 20.9 ± 7.5 (mg/kg.min), for vehicle and BVT116429 respectively.

### Effect on food intake, body weight and leptin

There were no differences in food intake between different treatment groups over the 10 day treatment period (Table 1). The group of rosiglitazone treated animals was the only group that gained weight during the study (Table 1). To determine the proportion of fat in the animals, fat pads were dissected and weighed and normalized to the whole body weight (fat pad/BW × 100, Table 1). The data showed no significant changes in fat mass after treatment with BVT116429 or rosiglitazone. In a separate experiment, body composition was determined by dual energy X-ray absorptimetry to take total body fat into account instead of epididymal fat alone. These results showed that there were no significant change in total body adiposity after treatment with BVT116429 compared to vehicle (vehicle, 36.2 ± 2.5% fat; 3 mg/kg, 38.2 ± 3.1%, 10 mg/kg, 36.9 ± 3.1 and 30 mg/kg, 37.6 ± 2.4%, n = 8 per group). By contrast, animals treated with rosiglitazone showed increased adiposity to 40.3 ± 3.4% (p < 0.01 versus vehicle). As expected, the leptin level was elevated for the rosiglitazone group compared with the vehicle group (Table 1). The highest dose group of BVT116429 (30 mg/kg) had higher leptin levels than the control group despite no significant changes in total body adiposity.

**Table 1:** Effect of 10-day treatment on various metabolic parameters

	Vehicle	3 mg/kg	10 mg/kg	30 mg/kg	Rosiglitazone
Food Intake (g)	47.8 ± 2.2	48.6 ± 2.3	51.1 ± 1.9	42.9 ± 1.3	50.6 ± 1.2
Body Weight (g)	39.9 ± 0.7	42.9 ± 1.2	41.2 ± 1.0	43.5 ± 1.3	45.0 ± 1.0**
Body Weight as percent of initial (%)	96.4 ± 1.0	98.0 ± 0.7	98.1 ± 0.5	100.4 ± 0.1	107.3 ± 1.2**
Initial body weight (g)	41.4 ± 0.8	43.8 ± 1.2	41.9 ± 0.8	43.3 ± 1.3	41.9 ± 0.68
Fat Pad weight (g)	1.60 ± 0.07	1.72 ± 0.05	1.55 ± 0.04	1.50 ± 0.07	1.68 ± 0.07
Fat pad/body weight ×100	4.02 ± 0.18	4.03 ± 0.19	3.77 ± 0.14	3.54 ± 0.23	3.74 ± 0.16
Leptin (ng/ml)	57.2 ± 0.7	68.6 ± 4.5	59.4 ± 2.3	73.6 ± 3.1*	83.6 ± 2.9***
Insulin (ng/ml)	20.8 ± 5.2	23.6 ± 4.0	13.6 ± 4.6	17.7 ± 8.4	11.8 ± 2.5
Triglycerides (ng/ml)	1.90 ± 0.18	2.29 ± 0.13	2.6 ± 0.46	3.03 ± 0.30	2.3 ± 0.29
Liver triglycerides (mg/g liver)	14.7 ± 1.0	20.4 ± 3.0	15.2 ± 1.0	18.5 ± 1.4	20.9 ± 2.0
Total cholesterol (mM)	4.0 ± 0.2	3.9 ± 0.1	4.0 ± 0.1	4.1 ± 0.2	5.1 ± 0.3**
HDL cholesterol (mM)	3.1 ± 0.2	3.0 ± 0.1	3.1 ± 0.1	3.2 ± 1.3	4.1 ± 0.2**
Liver cholesterol (mg/g liver)	4.0 ± 0.2	3.8 ± 0.2	4.3 ± 0.2	3.6 ± 0.1	3.2 ± 0.1*

Data are mean ± SEM, n = 8 per group. *p < 0.05, **p < 0.01 and ***p < 0.001 significantly different from vehicle

### Effect on plasma and liver lipids

Total plasma cholesterol was elevated in animals treated with rosiglitazone compared with the control group, and this was accompanied by elevated plasma HDL-cholesterol and lowered cholesterol in the liver (Table 1), but no effects were seen with BVT116429.

### Effects of the 11βHSD1 inhibitor BVT2733 on adiponectin concentrations

**Figure 8:**
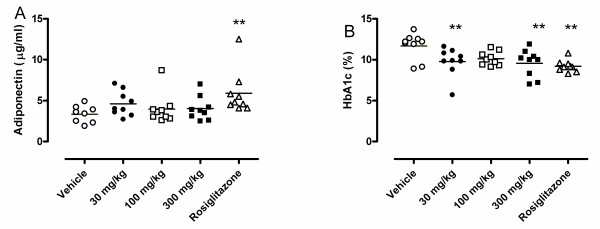
**Effects of BVT2733 on (A) adiponectin and (B) glycosylated hemoglobin in male KKA^y ^mice**. Animals were treated with vehicle (white circles), BVT2733 30 mg/kg (black circles), BVT2733 100 mg/kg (white squares), BVT2733 (black squares) or 5 mg/kg rosiglitazone (white triangles) for 8 days and plasma adiponectin and HbA1c were measured. * *p *< 0.05, ** *p *< 0.01, *** *p *< 0.001 versus vehicle, n = 9 per group.

To assess the effects of a second 11β HSD1 inhibitor, BVT2733, on plasma adiponectin concentrations, male KKA^y ^mice were treated with three different doses of the compound twice daily for 8 days, as described in the Methods section. Analysis of the plasma samples showed that BVT2733 had no significant effect on adiponectin concentrations (Fig. 8A). By contrast, rosiglitazone again caused significant increases in plasma adiponectin. Analysis of plasma HbA1c concentrations showed that the treatment with BVT2733 reduced HbA1c significantly at 30 and 300 mg/kg, confirming its expected anti-hyperglycemic effects (Fig. 8B).

## Discussion

Patients with type 2 diabetes have high intracellular levels of glucocorticoids [2]. The level is regulated by 11β HSD1 and inhibition of this enzyme in the adipose tissue is one possible novel way of treating type 2 diabetes. In contrast to their wild type siblings, 11β HSD1 -/- mice lack mRNA for 11β HSD1 and cannot convert 11-dehydrocorticosterone to active corticosterone [20]. When these mice were fed a high fat diet, blood glucose concentrations were lower compared with wild type animals. Indeed, 11β HSD1 -/- mice fed a high fat diet do not become hyperglycemic. Morton *et al *studied 11β HSD1 -/- mice with a focus on lipid metabolism and reported lower plasma triglyceride levels compared with wild type animals [19]. The same group studied mRNA expression of leptin and adiponectin in epididymal fat of 11β HSD1 -/- mice and saw decreased leptin and increased adiponectin [5]. Kershaw *et al *created a transgenic model in which human 11β HSD2 was expressed under the control of the murine adipocyte fatty acid binding protein promoter and thereby increased 11β HSD2 expression and activity exclusively in adipose tissue, resulting in a phenotype with less glucocorticoid in adipose tissue due to enhanced inactivation of corticosterone. This model displayed increased expression of adiponectin in subcutaneous adipose tissue, when fed a high fat diet compared with wild type [21]. This is in accordance with the report of Masuzaki *et al *which describes transgenic mice overexpressing 11β HSD1 in adipose tissue with decreased adiponectin expression in mesenteric fat [4]. Mesenteric fat was increased nearly four times in this animal and expression of mRNA of adiponectin was decreased to 42% compared to non-transgenic mice. Transgenic mice also had reduced glucose tolerance and insulin sensitivity, measured by intraperitoneal glucose tolerance tests and insulin tolerance tests, compared to non-transgenic mice.

Thus these three animal models implicate an association between glucocorticoid dynamics in adipose tissue and adiponectin expression. In the present study, we show that treatment with the specific 11β HSD1 inhibitor BVT116429 (30 mg/kg) increases adiponectin concentrations, both in adipocytes and, quantitatively and qualitatively, in serum. This is in accordance with other studies where glucocorticoid is reported to inhibit both expression and secretion of adiponectin [6,28]. Adiponectin is an activator of AMP activated protein kinase (AMPK) which is proposed to be a metabolic master switch [29]. Activation of AMPK is supposed to lead to improved glucose and fat metabolism including increased glucose uptake in muscle, increased insulin sensitivity, increased FFA uptake and oxidation and repressed lipogenesis [30,31]. Interestingly some of these ameliorations are achieved by treatment with BVT116429. In this study we show that treatment with the specific 11β HSD1 inhibitor BVT116429 (30 mg/kg) improves the glucose homeostasis in diabetic KKA^y ^mice. Fasting blood glucose was lowered by approximately 25% (Fig. 4) after ten days and plasma insulin was reduced already after seven days of treatment. Unfortunately the ITT did not show any significant differences in insulin sensitivity between the groups, probably due to a combination of groups of too few animals in which insulin resistance varies substantially, and a varied response to the experimental dose of insulin. 11β HSD1 -/- mice have higher expression of uncoupling protein 2 (UCP-2) when fed high fat diet and this might be due to the elevated expression in adiponectin [5]. Similar metabolic modifications were shown with another 11β HSD inhibitor, compound 544. Fasting serum glucose was lowered by 15% and insulin levels were normalised in diet induced obese mice after 11 days treatment [25]. The same group also reported decreased food intake and decreased body weight gain.

Adiponectin is an important link between type 2 diabetes and vascular disease[32,33]. Kumada *et al *showed in their study in men that hypoadiponectinemia was associated with coronary artery disease prevalence even after adjustment for risk factors such as type 2 diabetes and body mass index [34]. Adiponectin suppresses atherogenesis by inhibiting adherence of monocytes, reducing their phagocytic activity and suppressing accumulation of lipoproteins in the vascular wall [35,36]. Hermanowski-Vosatka *et al *[25] showed that inhibition with compound 544, another 11β HSD inhibitor, slowed plaque progression in a mouse model of atherosclerosis. We suggest that the beneficial results regarding atherosclerosis may be due, at least in part, to increased adiponectin. This study did not report on concentrations of adiponectin in 544-treated animals but we hypothesize that prolonged treatment with BVT116429 will demonstrate similar effects due to elevations in circulating adiponectin.

Our earlier 11β HSD1 inhibitor, BVT2733, has been shown to exert significant effects on feeding behavior and energy balance as well as glucose homeostasis in various animal models of glucose intolerance and type 2 diabetes [22-24]. Thus, the compound has shown strong effects in the liver and possibly in the brain. Our analysis here shows that BVT2733 has no effect on plasma adiponectin concentrations, whilst BVT116429 has no effects on the hepatic glucose production. These data indicate therefore that whilst inhibition of 11β HSD1 can be expected to be beneficial for treating the pathology of type 2 diabetes mellitus, all inhibitors are not the same, and different mechanistic effects can be expected with different compounds. The differences likely lie in the subtly different pharmacodynamics exerted by the compounds in different tissues in the body. Thus BVT2733, which has strong effects in the liver, likely regulates glucose homeostasis in this organ, whilst BVT116429 has less effect in the liver but upregulates the major insulin sensitizing adipokine adiponectin, due to its greater effects in the adipose tissue.

## Conclusion

In our study we have treated an animal model of type 2 diabetes with an 11β HSD1 inhibitor BVT116429 for up to ten days and obtained several ameliorations of the diabetic phenotype, specifically lowered plasma glucose, which is the most important clinical chemistry parameter to improve. Ten days is a relatively short treatment time and a longer treatment could possibly lead to further improvements such as improved dyslipidemia. Increases in adiponectin concentrations may be an integral component in the mechanism of action of 11β HSD1 inhibitors which are active in the adipose tissue and may be a useful marker of efficacy during the clinical development of inhibitory compounds. This approach to the treatment of type 2 diabetes might also reduce the risk of cardiovascular disease.

## Methods

### Animals

Male mice, KK-A^y ^(KKA^y^), from Charles River (Tokyo, Japan) were given a high fat diet (D12266B, 32.5% fat, Research Diets, NJ, USA) and water *ad libitum*. Animals were kept in individual cages under a 12-h light/12-h dark cycle, at 22 ± 1°C and 50% humidity. KKA^y ^mice were randomized into groups. One set of animals were administered 11β HSD-inhibitor BVT116429 (3, 10 or 30 mg/kg), or rosiglitazone (5 mg/kg) or vehicle by oral gavage (p.o.) once daily at 1700 for 10 days. The other set of animals were administered 11β HSD-inhibitor BVT2733 (30, 100 or 300 mg/kg), rosiglitazone (5 mg/kg) or vehicle by oral gavage twice daily at 0700 and 1700 for eight days. Body weight and food intake were monitored at the beginning and at the end of the study. Body composition was analyzed by dual energy X-ray absorptimetry using a Lunar Piximus densitometer. Terminal blood samples for plasma analysis were taken after four hours of fasting at 1200 from the orbital sinus or via heart puncture in anaesthetized animals prior to euthanasia. The procedures involving animals were in conformity with national and international laws for the care and use of laboratory animals and were approved by the local animal ethical committee.

### Oral glucose tolerance test (oGTT)

An oral glucose tolerance test was performed after an overnight fast and two hours post-dosing on day seven of the 10-day experiment. Mice were given an oral dose of glucose (2 g/kg; 200 mg/ml stock solution, Fresenius Kabi, Uppsala, Sweden) and blood samples (tail cut, 25 μl) for glucose (Accu-check, Roche, USA) and insulin (ELISA, Mercodia, Uppsala, Sweden) were taken immediately before the glucose load and 0, 15, 30, 60 and 120 min after the glucose load.

### Insulin tolerance test (ITT)

An insulin tolerance test was performed after a four hour fast on day seven. Two hours after the daily dose of substance or vehicle, mice were given an intraperitoneal dose of insulin (1 U/kg) and blood samples (tail cut, 5 μl) for analyses of glucose (Accu-check, Roch, USA) were taken after 0, 15, 30 60 and 120 min. For data analysis, glucose concentrations were normalized to the concentration at time zero and the rate of glucose elimination was calculated by fitting the data to a single exponential equation (y = y_o_e^-kt^).

### Euglycemic hyperinsulinemic clamp

Catheters were placed into the right jugular vein of male KKA^y ^mice under anesthesia, and the animals were allowed to recover for three days. After dosing with vehicle or BVT116429 (30 mg/kg, p.o.) once daily for eight days, animals were fasted for 6 hours. At the start of the clamp experiment, all animals were infused with radiolabelled glucose tracer (2.5 μCi/kg/min) and 70 minutes later, insulin infusion was started at a rate of 12.5 mU/kg/min. Blood glucose concentrations were monitored thereafter and glucose was infused from a 30% solution as required to maintain euglycemia. The experiment was terminated after 160 minutes (90 minutes after insulin infusion was commenced) and animals were euthanized. Data from animals were included in the final analysis if there was no more than 20% variation in basal and clamped glucose levels, if there was at least a two-fold increase in blood insulin concentrations upon insulin infusion, if there was a specific activity of tracer glucose of at least 40000 cpm per mg glucose in whole blood and if the catheter was shown to be correctly inserted when the experiment was terminated. Glucose disposal rate (GDR) was calculated as (cpm/min) infused/(cpm/mg glucose in blood). Under basal conditions prior to clamp, endogenous glucose production (EGP) equals GDR. Under clamped conditions, EGP was calculated as the difference between GDR and glucose infusion rate (GIR).

### Clinical chemistry

Blood samples were taken from anesthetized (Forene, Abbot, Chicago, USA) mice via heart puncture or orbital plexus, after a four hour fast on the final day. Samples were kept on ice for 30 min before centrifugation at 2000–3000 g for 10 min and subsequently stored in tubes at -70°C until analysis. Glucose concentrations were analyzed with a UV method (Hitachi 912 Multianalyzer, Roche Diagnostics, Switzerland). HbA1c, cholesterols, triglycerides and free fatty acids were analyzed with an enzymatic colorimetric method (Hitachi 912 Multianalyzer, Roche Diagnostics, Switzerland). Serum adiponectin and leptin were analyzed with radioimmuno assays (Linco Research, St.Louis, MO, USA). Serum insulin was analyzed using an enzyme-linked immunoassay (ELISA, Mercodia, Uppsala, Sweden).

### Tissues

The epididymus fat pad and liver were dissected, weights were measured and tissue samples were snap frozen in liquid nitrogen and stored at -70°C. Lipid extracts were prepared by homogenizing frozen liver in Heptan:Isopropanol 3:2, Tween 1%. (1:10) and then analyzed with an enzymatic colorimetric test (Roche Diagnostic, Switzerland).

### 11β-HSD1 ex vivo Assay

DMEM (GIBCO BRL, 11965-092) media (37°C) was supplemented with NADPH (Sigma, Cat# N1630) and cortisone (Sigma, Cat# C2755) to a final concentration of 100 μM and 1μM, respectively. 500 μl supplemented media was dispensed to each well of a 24-well plate (Falcon, BD, Cat# 353047). Frozen tissue was dissected into 30–40 mg aliquots (weights recorded) and directly placed in the pre-warmed media. Plates were incubated at 37°C for 3 hours with substrate, and media subsequently collected (without the tissue pieces) by transferring the supernatant to a fresh 24-well plate (samples stored at -80°C until ready for assay). Cortisol ELISA (Correlate-EIA kit, Assay Designs Inc. Cat# 901-071) was performed as suggested by the manufacturer with appropriate sample dilution (usually 1:10 for mouse adipose tissue).

### Velocity sedimentation of serum samples

5–40% sucrose gradients in 10 mM HEPES, pH 8.0, 125 mM NaCl were poured stepwise in 2 ml thin walled ultracentrifuge tubes (Becton-Dickinson) and allowed to equilibrate overnight at 4°C. Following layering of the sample on top (diluted 1:10 with 10 mM HEPES, pH 8.0, 125 mM NaCl), gradients were spun at 55 000 rpm for 4 hours at 4°C in a Beckman Optima XL-80K ultracentrifuge (SW55TI). 200 μl gradient fractions were sequentially retrieved and analyzed by Western blot.

### Immunoblotting

Proteins were separated by SDS-PAGE (non-reducing, non-heat denaturing) and subsequently transferred to PVDF-membranes (BioRad). Membranes were blocked with 5% non-fat dry milk in TBST (Tris-buffered saline, 0.1% Tween20). As primary antibody, a rabbit polyclonal antibody against adipose-denied complement-related protein (Acrp30) was used (Santa Cruz #sc-17044-R). As secondary antibody, a horseradish peroxidase (HRP)-conjugated goat anti-rabbit antibody was used (Upstate #12-348). Blots were visualized using chemiluminiscense, (enhanced chemiluminiscense (ECL) Plus detection kit, GE Healthcare).

### Adiponectin analyses in human primary adipocytes

Pre-adipocytes (Body mass index <25) were purchased from ZenBio (SP-F-1). Culture medium (PM-1), differentiation medium (DM-2) and adipocyte medium (AM-1) were also from ZenBio. One ampoule of human primary pre-adipocytes was thawed in a 37°C water bath and subsequently suspended in 10 ml culture medium. 30 μl of the cell suspension was transferred to an Eppendorf tube and 15 μl Trypan blue was added. Cells were counted and the viability was calculated. The remaining cell suspension was diluted to a final volume of 78 ml culture medium. Cell suspension aliquots, containing 8–9000 cells, were added per well in 60 wells of a 96-well plate. Cells were differentiated according to the ZenBio-protocol. On the 11th day from the start of differentiation (before the peak of adiponectin secretion), dexamethasone was removed from the medium. 24 hours later, the cells were subjected to treatments as follows: medium only, 100 nM cortisone, 0.1 μM BVT116429 + 100 nM cortisone, 10 μM BVT116429 + 100 nM cortisone. Cells were exposed for 48 hours where after the medium was transferred to a fresh 96-well plate and stored at -20°C until analyzed. The adiponectin levels were analyzed with a human adiponectin ELISA kit (CYT350, Chemicon Int.) according to the manufacturer's instructions.

### Statistical analysis

Data are expressed as means ± SE. Results were subjected to a one-way ANOVA followed by Dunett's multiple comparison test, or the nonparametric Kruskal-Wallis test followed by Dunn's test. Prism 4 for windows (GraphPad software Inc) was used for statistical analysis.

## Authors' contributions

CK is responsible for the analysis and interpretation of the different adiponectin complex in serum and measurements of adiponcetin in adipose tissue. CK has been involved in writing the manuscript. EB contributed to the design of the study and has critically revised it. VC carried out the 11β-HSD ex vivo assay studies and has critically revised the manuscript. CL analyzed the clinical chemistry and has critically revised the manuscript. GS and CSN were responsible for the clamp-study and have critically revised the manuscript. SJ has contributed to the design, interpreted data and been involved in writing the manuscript. MS has designed, carried out and interpreted the in vivo parts and has been involved in writing the manuscript. All authors have read and approved the final manuscript.

## References

[B1] Friedman TC, Mastorakos  G, Newman  TD, Mullen NM, Horton EG, Costello R, Rapadopoulos  NM, Chrousos GP (1996). Carbohydrate and lipid metabolism in endogenous hypercortisolism: shared features with metabolic syndrome X and NIDDM.. Endocr J.

[B2] Rask E, Olsson T, Soderberg S, Andrew R, Livingstone DE, Johnson O, Walker BR (2001). Tissue-specific dysregulation of cortisol metabolism in human obesity. J Clin Endocrinol Metab.

[B3] Almelung  D, Huebener HJ, Roka L, Meyerheim  G (1953). Conversion of cortisone to compound F.. J Clin Endocrinol Metab.

[B4] Masuzaki H, Paterson J, Shinyama H, Morton NM, Mullins JJ, Seckl JR, Flier JS (2001). A transgenic model of visceral obesity and the metabolic syndrome. Science.

[B5] Morton NM, Paterson JM, Masuzaki H, Holmes MC, Staels B, Fievet C, Walker BR, Flier JS, Mullins JJ, Seckl JR (2004). Novel adipose tissue-mediated resistance to diet-induced visceral obesity in 11 beta-hydroxysteroid dehydrogenase type 1-deficient mice. Diabetes.

[B6] Fallo F, Scarda A, Sonino N, Paoletta A, Boscaro M, Pagano C, Federspil G, Vettor R (2004). Effect of glucocorticoids on adiponectin: a study in healthy subjects and in Cushing's syndrome. Eur J Endocrinol.

[B7] Hotta K, Funahashi T, Arita Y, Takahashi M, Matsuda M, Okamoto Y, Iwahashi H, Kuriyama H, Ouchi N, Maeda K, Nishida M, Kihara S, Sakai N, Nakajima T, Hasegawa K, Muraguchi M, Ohmoto Y, Nakamura T, Yamashita S, Hanafusa T, Matsuzawa Y (2000). Plasma concentrations of a novel, adipose-specific protein, adiponectin, in type 2 diabetic patients. Arterioscler Thromb Vasc Biol.

[B8] Lara-Castro C, Luo N, Wallace P, Klein RL, Garvey WT (2006). Adiponectin multimeric complexes and the metabolic syndrome trait cluster. Diabetes.

[B9] Pajvani UB, Hawkins M, Combs TP, Rajala MW, Doebber T, Berger JP, Wagner JA, Wu M, Knopps A, Xiang AH, Utzschneider KM, Kahn SE, Olefsky JM, Buchanan TA, Scherer PE (2004). Complex distribution, not absolute amount of adiponectin, correlates with thiazolidinedione-mediated improvement in insulin sensitivity. J Biol Chem.

[B10] Kadowaki T, Yamauchi T (2005). Adiponectin and adiponectin receptors. Endocr Rev.

[B11] Mao X, Kikani CK, Riojas RA, Langlais P, Wang L, Ramos FJ, Fang Q, Christ-Roberts CY, Hong JY, Kim RY, Liu F, Dong LQ (2006). APPL1 binds to adiponectin receptors and mediates adiponectin signalling and function. Nat Cell Biol.

[B12] Hu E, Liang P, Spiegelman BM (1996). AdipoQ is a novel adipose-specific gene dysregulated in obesity. J Biol Chem.

[B13] Spranger J, Kroke A, Mohlig M, Bergmann MM, Ristow M, Boeing H, Pfeiffer AF (2003). Adiponectin and protection against type 2 diabetes mellitus. Lancet.

[B14] Yamauchi T, Kamon J, Waki H, Terauchi Y, Kubota N, Hara K, Mori Y, Ide T, Murakami K, Tsuboyama-Kasaoka N, Ezaki O, Akanuma Y, Gavrilova O, Vinson C, Reitman ML, Kagechika H, Shudo K, Yoda M, Nakano Y, Tobe K, Nagai R, Kimura S, Tomita M, Froguel P, Kadowaki T (2001). The fat-derived hormone adiponectin reverses insulin resistance associated with both lipoatrophy and obesity. Nat Med.

[B15] Maeda N, Takahashi M, Funahashi T, Kihara S, Nishizawa H, Kishida K, Nagaretani H, Matsuda M, Komuro R, Ouchi N, Kuriyama H, Hotta K, Nakamura T, Shimomura I, Matsuzawa Y (2001). PPARgamma ligands increase expression and plasma concentrations of adiponectin, an adipose-derived protein. Diabetes.

[B16] Yu JG, Javorschi S, Hevener AL, Kruszynska YT, Norman RA, Sinha M, Olefsky JM (2002). The effect of thiazolidinediones on plasma adiponectin levels in normal, obese, and type 2 diabetic subjects. Diabetes.

[B17] Nawrocki AR, Rajala MW, Tomas E, Pajvani UB, Saha AK, Trumbauer ME, Pang Z, Chen AS, Ruderman NB, Chen H, Rossetti L, Scherer PE (2006). Mice lacking adiponectin show decreased hepatic insulin sensitivity and reduced responsiveness to peroxisome proliferator-activated receptor gamma agonists. J Biol Chem.

[B18] Yang B, Brown KK, Chen L, Carrick KM, Clifton LG, McNulty JA, Winegar DA, Strum JC, Stimpson SA, Pahel GL (2004). Serum adiponectin as a biomarker for in vivo PPARgamma activation and PPARgamma agonist-induced efficacy on insulin sensitization/lipid lowering in rats. BMC Pharmacol.

[B19] Morton NM, Holmes MC, Fievet C, Staels B, Tailleux A, Mullins JJ, Seckl JR (2001). Improved lipid and lipoprotein profile, hepatic insulin sensitivity, and glucose tolerance in 11beta-hydroxysteroid dehydrogenase type 1 null mice. J Biol Chem.

[B20] Kotelevtsev Y, Holmes MC, Burchell A, Houston PM, Schmoll D, Jamieson P, Best R, Brown R, Edwards CR, Seckl JR, Mullins JJ (1997). 11beta-hydroxysteroid dehydrogenase type 1 knockout mice show attenuated glucocorticoid-inducible responses and resist hyperglycemia on obesity or stress. Proc Natl Acad Sci U S A.

[B21] Kershaw EE, Morton NM, Dhillon H, Ramage L, Seckl JR, Flier JS (2005). Adipocyte-specific glucocorticoid inactivation protects against diet-induced obesity. Diabetes.

[B22] Alberts P, Engblom L, Edling N, Forsgren M, Klingstrom G, Larsson C, Ronquist-Nii Y, Ohman B, Abrahmsen L (2002). Selective inhibition of 11beta-hydroxysteroid dehydrogenase type 1 decreases blood glucose concentrations in hyperglycaemic mice. Diabetologia.

[B23] Alberts P, Nilsson C, Selen G, Engblom LO, Edling NH, Norling S, Klingstrom G, Larsson C, Forsgren M, Ashkzari M, Nilsson CE, Fiedler M, Bergqvist E, Ohman B, Bjorkstrand E, Abrahmsen LB (2003). Selective inhibition of 11 beta-hydroxysteroid dehydrogenase type 1 improves hepatic insulin sensitivity in hyperglycemic mice strains. Endocrinology.

[B24] Wang SJ, Birtles S, de Schoolmeester J, Swales J, Moody G, Hislop D, O'Dowd J, Smith DM, Turnbull AV, Arch JR (2006). Inhibition of 11beta-hydroxysteroid dehydrogenase type 1 reduces food intake and weight gain but maintains energy expenditure in diet-induced obese mice. Diabetologia.

[B25] Hermanowski-Vosatka A, Balkovec JM, Cheng K, Chen HY, Hernandez M, Koo GC, Le Grand CB, Li Z, Metzger JM, Mundt SS, Noonan H, Nunes CN, Olson SH, Pikounis B, Ren N, Robertson N, Schaeffer JM, Shah K, Springer MS, Strack AM, Strowski M, Wu K, Wu T, Xiao J, Zhang BB, Wright SD, Thieringer R (2005). 11beta-HSD1 inhibition ameliorates metabolic syndrome and prevents progression of atherosclerosis in mice. J Exp Med.

[B26] Fotsch C, Askew BJ, Chen JJ (2005). 11beta-Hydroxysteroid dehydrogenase-1 as therapeutic target for metabolic diseases.. Expert Opin Ther Patents.

[B27] St Jean DJ, Yuan C, Bercot EA, Cupples R, Chen M, Fretland J, Hale C, Hungate RW, Komorowski R, Veniant M, Wang M, Zhang X, Fotsch C (2007). 2-(S)-phenethylaminothiazolones as potent, orally efficacious inhibitors of 11beta-hydroxysteriod dehydrogenase type 1. J Med Chem.

[B28] Zhang Y, Matheny M, Zolotukhin S, Tumer N, Scarpace PJ (2002). Regulation of adiponectin and leptin gene expression in white and brown adipose tissues: influence of beta3-adrenergic agonists, retinoic acid, leptin and fasting. Biochim Biophys Acta.

[B29] Winder WW, Hardie DG (1999). AMP-activated protein kinase, a metabolic master switch: possible roles in type 2 diabetes. Am J Physiol.

[B30] Merrill GF, Kurth EJ, Hardie DG, Winder WW (1997). AICA riboside increases AMP-activated protein kinase, fatty acid oxidation, and glucose uptake in rat muscle. Am J Physiol.

[B31] Sullivan JE, Brocklehurst KJ, Marley AE, Carey F, Carling D, Beri RK (1994). Inhibition of lipolysis and lipogenesis in isolated rat adipocytes with AICAR, a cell-permeable activator of AMP-activated protein kinase. FEBS Lett.

[B32] Ouchi N, Shibata R, Walsh K (2006). Cardioprotection by adiponectin. Trends Cardiovasc Med.

[B33] Ekmekci H, Ekmekci OB (2006). The role of adiponectin in atherosclerosis and thrombosis. Clin Appl Thromb Hemost.

[B34] Kumada M, Kihara S, Sumitsuji S, Kawamoto T, Matsumoto S, Ouchi N, Arita Y, Okamoto Y, Shimomura I, Hiraoka H, Nakamura T, Funahashi T, Matsuzawa Y (2003). Association of hypoadiponectinemia with coronary artery disease in men. Arterioscler Thromb Vasc Biol.

[B35] Matsuzawa Y, Funahashi T, Kihara S, Shimomura I (2004). Adiponectin and metabolic syndrome. Arterioscler Thromb Vasc Biol.

[B36] Ouchi N, Kihara S, Arita Y, Maeda K, Kuriyama H, Okamoto Y, Hotta K, Nishida M, Takahashi M, Nakamura T, Yamashita S, Funahashi T, Matsuzawa Y (1999). Novel modulator for endothelial adhesion molecules: adipocyte-derived plasma protein adiponectin. Circulation.

